# Analysis of Apple Candying by Microwave Spectroscopy

**DOI:** 10.3390/foods8080316

**Published:** 2019-08-04

**Authors:** Juan A. Tomas-Egea, Pedro J. Fito, Marta Castro-Giraldez

**Affiliations:** Instituto Universitario de Ingeniería de Alimentos para el Desarrollo, Universitat Politècnica de València, Camino de Vera s/n, 46022 Valencia, Spain

**Keywords:** dielectric spectroscopy, permittivity, dehydration, candying, hot air drying, isotherms, sucrose

## Abstract

Process control in the industry requires fast, safe and easily applicable methods. In this sense, the use of dielectric spectroscopy in the microwave range can be a great opportunity to monitor processes in which the mobility and quantity of water is the main property to produce a quality and safety product. The candying of fruits is an operation in which the samples are first osmotically dehydrated and then exposed to a hot air-drying operation. This process produces changes in both the structure of the tissue and the relationships between water, the solid matrix and the added soluble solids. The aim of this paper is to develop a dielectric tool to predict the water/sucrose states throughout the candying of apple, by considering the complexity of the tissue and describing the different transport phenomena and the different transition processes of the sucrose inside the sample.

## 1. Introduction

Dehydration is probably one of the most important methods of foods preservation. The main objective in dehydrating agricultural products is the removal of water in the foods up to a certain level of water activity, at which microbial spoilage and deterioration chemical reactions are minimized [1]. Apples are consumed either fresh or in the form of various processed products such as juice, jam, marmalade and dried products [2], being the dried apples in extensive demand. Candying is an industrial operation, which consists on osmotic dehydration (OD) followed by a hot-air drying (HAD) treatment. OD consists of the immersion of foods in hypertonic solutions with the objective of producing a water flow out of the food and a simultaneous flow of solutes inside the tissue. In fruits, these mass transfer phenomena affect the apple structure. Fruits are formed by vegetal cells, conforming the parenchyma tissue, which is a complex structure with intercell connections (plasmodesmata), intracellular and extracellular spaces [3,4,5]. Intracellular volume is mainly occupied by vacuoles, which are fundamentally a water solution with multiple solutes. The membrane of the cell is named protoplast, it is selectively permeable (active and passive protein channels) and controls turgor and the cell growth [6]. The cell membrane has protein channels named aquaporin and calcium protein channel that are responsible of the water transport and the cell homeostasis [7]. The cell wall provides mechanical resistance to the cell. In the union between cell walls and protoplast are bonds of Na^+^, protein microtubules and conduits named plasmodesmata that allow the transport between the adjacent cells (symplastic pathways). Extracellular volume comprehends the cell wall and the spaces between cells [3]. OD produces different phenomena in the cellular tissue, first dehydration with shrinkage and solute intake in extracellular space. Continuous shrinkage produces a breakdown between wall and protoplast called plasmolysis causing mechanical driving forces (swelling/shrinkage) [8,9,10,11]. The changes in cellular tissue affect the water mobility and its distribution [12].

HAD influences on fruit quality because it produces chemical, physical and biological changes in food [13]. Moreover, the internal structure undergoes deformation and could be locally damaged. Removal of water adds rigidity to the external layers and simultaneously builds up moisture gradients, which create shrinkage stresses [14]. The understanding of these effects is still limited, and more studies are necessary in this field in order to improve food quality and nutritional characteristics of dehydrated foods.

During the candying process, crystallization of sucrose occurs. Sucrose crystallizes in supersaturated aqueous solutions. The sucrose molecule has eight hydroxyl groups, which can be involved in hydrogen bond formation. In sufficiently diluted aqueous solutions, all the hydroxyl groups form hydrogen bonds with water molecules. If the concentration increases, the molecules start to interact forming an intramolecular bond and then two intermolecular bonds [15]. If sucrose concentration in the solution increases, aggregation phenomena occur between sucrose molecules, leading to stable three-dimensional nucleus [16]. After nucleus formation, the crystal growth consists in the incorporation of sucrose molecules to the crystal lattice, which requires the migration of hydration water from the crystal surface to the solution [17]. 

The analysis of the electromagnetic field (EMF) properties in range of microwaves could be a good tool to monitor and improve apple candying. The development of sensors to determine the dielectric properties of biological tissues, in range of radiofrequency and microwaves, has been demonstrated to be a useful tool for monitoring the quality of many products: Poultry [18,19,20,21,22], pork [23,24,25,26,27], beef [28], goat [29], meat products [30], cheese [31], mandarin [32], potato [33], wheat [34], agricultural products [35,36,37], pomegranate [38] and apple [39,40,41], and for monitoring processes of pork meat HAD [42] and salting [43], orange peel drying [44], cheese salting [45], brewing [46], puffing of amaranth seeds [47], OD of kiwi [48] and apple [49].

The EMF is a flux of photons [50] and the interaction with matter can be modeled by Schrodinger’s equation [51] attending to the quantum theory. However, at the macroscopic level, it is possible to apply the Maxwell’s equations [52], where the physical property that describes the electric effect is the complex permittivity and for the magnetic effect is the complex permeability [50]. In the microwave range (1.24·10^−6^ to 1.24·10^−3^ eV or 300 MHz to 300 GHz), these interactions are described by γ-dispersion and ionic conductivity. γ-dispersion is caused by the induction and orientation of dipolar molecules, being water the most important in biological systems [53]. These phenomena generate electric energy accumulation caused mainly by water spin reorientation and it is represented by the real part of the permittivity (ε′). On the other hand, a part of the electrical energy of photons is transformed in other energies (mechanical or calorific) due to the collisions or frictions associated to the increase of molecular mobility; this part of the electric energy is called the loss factor (ε″). In the microwave range, the vibration of chemical species with very high ionic strength causes a part of the losses of electrical energy; this is called ionic conductivity (σ) [54].

The aim of this paper is to develop a dielectric tool to predict the water/sucrose states throughout the candying of apple, by considering the complexity of the tissue and describing the different transport phenomena and the different transition processes of the sucrose inside the sample.

## 2. Materials and Methods 

**Figure 1:**
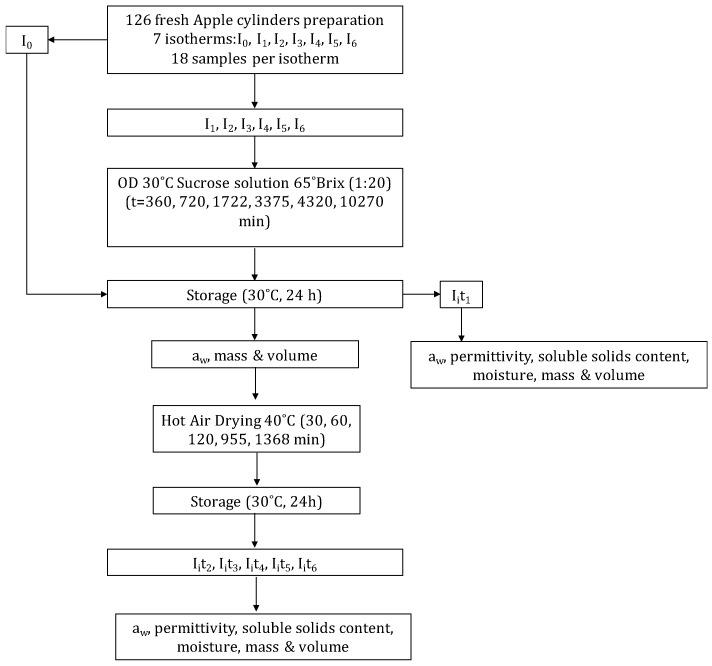
Flow diagram of the experimental procedure, where I represents each isotherm, subindices i from 0 to 6 of each isotherm represent a concrete sucrose/solid matrix weight relation, t_1_ to t_6_ represent the different times of hot-air drying (HAD).

Apples (var. Granny Smith) were bought from a local supermarket and kept refrigerated until use. The apples were cut with a caliper and a cork borer in cylinders (1 cm thickness, 2 cm diameter) from the parenchymatic tissue. There were prepared 126 samples in order to obtain 7 isotherms (Figure 1). Eighteen samples were used to obtain the isotherm of raw apple (I_0_): Three samples to characterize the raw material, it is without hot air dehydration, and 15 exposed to hot air dehydration. The remaining 108 samples were dehydrated osmotically to obtain six isotherms (I_1_ to I_6_); 18 samples were used for each selected time of OD: Three samples to characterize osmotic dehydrated samples, it is without hot air dehydration, and 15 exposed to hot air dehydration. In conclusion, seven isotherms were obtained, considering that the samples of an isotherm have the same solid matrix/sucrose weight relation.

Sucrose solution (65% *w*/*w*, 30 °C), prepared with commercial sugar and distilled water, was used as an osmotic agent. The relation between the fruit and the osmotic solution was of 1:20 (*w*/*w*) to avoid changes in the solution during the process. The system was maintained at 30 °C in a constant-temperature chamber. To prevent evaporation the vessel was covered with a sheet of plastic wrap. Preliminary kinetic studies were done at the same working conditions in order to select the OD treatment times. The OD treatment times in the preliminary studies were: 180, 360, 720, 1463, 1577, 1722, 3375, 4320, 7200, 8640, 10270, 14590 and 23230 min. Based on the results [54], OD treatment times were selected for this research: 0, 360, 720, 1722, 3375, 4320 and 10270 min. After the treatment, the samples were removed from the solution and blotted with a paper to remove the superficial osmotic solution. Then, the samples were kept at 30 °C for 24 h, on AquaLab disposable sample containers, closed with parafilm^®^. The mass, volume and water activity of the 126 samples were measured after the repose. Moreover, permittivity, moisture and soluble solids content (°Brix) of three samples of each OD treatment time were measured to characterize each sucrose/solid matrix weight relation (x_S_/x_SM_), considering as the solid matrix mass is neither water nor solutes. The remaining samples in each isotherm were hot air dried (times of HAD treatment: 30, 60, 120, 955 and 1368 min). Three samples were used in each HAD treatment time. The drying experiments were carried out at 40 °C drying air temperature. The convective dryer was designed and built in the Food Technology Department of Universitat Politècnica de València, has a control unit for setting the velocity and temperature of air, which is heated through electrical resistances. Air velocity was kept at a constant value of 1.5 ± 0.2 m s^−1^ in all experiments.

After the drying treatment, samples were maintained at 30 °C for 24 h, on AquaLab disposable sample containers, closed with parafilm^®®^. After this repose time, the permittivity, mass, volume, water activity, moisture and soluble solids content were measured.

Volume measurements were analyzed by image analysis and the software Adobe Photoshop^®®^ CS5 (Adobe Systems Inc., San Jose, CA, USA) to get the diameter and the thickness of the samples. The images of the samples were obtained with a digital camera (Canon EOS 550D, with a size of 2592 × 1728 pixels and a resolution of 16 pixel/mm). 

Mass was determined by using a Mettler Toledo Balance (±0.0001 g; Mettler-Toledo, Inc., Columbus, OH, USA). Measurements were done in structured samples (not minced), thus the obtained a_w_ is considered to be the surface a_w_ [54].

Water activity was measured in the structured samples with a dew point hygrometer Decagon (Aqualab^®®^ series 3TE) with precision ±0.003.

Moisture content in the apple cylinders was determined gravimetrically at 60 °C in a vacuum oven until constant weight was reached [55]. Sugar content was determined in a refractometer (ABBE, ATAGO Model 3-T, Tokyo, Japan).

The system used to measure permittivity consists of an Agilent 85070E open-ended coaxial probe (Agilent, Santa Clara, CA, USA) connected to an Agilent E8362B Vector Network Analyzer (Agilent, Santa Clara, CA, USA). The system was calibrated using three different types of loads: Open (air), short-circuit and 30 °C Milli-Q water. Once the calibration was carried out, 30 °C Milli-Q water was measured again to check calibration suitability. Permittivity was measured from 500 MHz to 20 GHz. The measurements were performed in triplicate. Dielectric constant (ε′) was modeled adjusting the experimental data using Traffano-Schiffo model [20] (Equation (1)) in order to obtain information of γ-dispersion:

(1) $$log\varepsilon^{\prime }\left(\omega \right)=log{\varepsilon^{\prime }}_{\infty }+\sum_{n=1}^{3}\frac{\Delta log\varepsilon_{n}^{\prime }}{1+e^{\left(log\omega^{2}-log\tau_{n}^{2}\right)\cdot \alpha_{n}}},$$

where n represents α, β or γ dispersion, $log\varepsilon^{\prime }$ represents the decimal logarithm of the dielectric constant, $log{\varepsilon^{\prime }}_{\infty }$ the logarithm of the dielectric constant at high frequencies, logω represents the decimal logarithm of the angular velocity (obtained from the frequency), $\Delta log{\varepsilon^{\prime }}_{n}$ ($\Delta l{\varepsilon^{\prime }}_{n}\;$= $\log{\varepsilon^{\prime }}_{n}-\log{\varepsilon^{\prime }}_{n-1})$ the amplitude of the n dispersion, $log\tau_{n}$ the logarithm of the angular velocity at relaxation time for each n dispersion and $\alpha_{n}$ are the dispersion slopes. In this work, this model was applied for γ-dispersion only. 

## 3. Results

**Figure 2:**
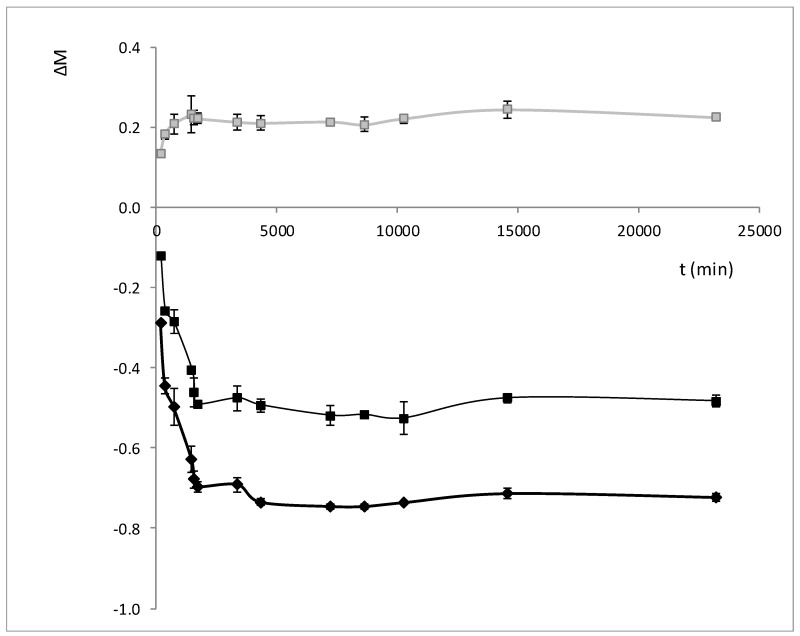
Evolution of overall mass (**■**), water mass (**♦**) and sucrose mass (**■**) through the osmotic treatment.

In order to understand the mechanisms that govern the relationship between water and sucrose in plant tissue matrix during OD and HAD treatments, and thus develop dielectric predictive tools that not only explain the water state but also explain the state of the whole internal liquid phase of the vegetal tissue, a kinetic analysis of the variation of overall mass, water and sucrose was proposed as a first step. Overall mass, water and sucrose mass variations throughout the OD treatment are shown in Figure 2. These parameters were estimated by Equations (2)–(4), respectively.

(2) $$\Delta M=\frac{M_{t}-M_{0}}{M_{0}},$$

(3) $$\Delta M_{w}=\frac{M_{t}x_{wt}-M_{0}x_{w0}}{M_{0}},$$

(4) $$\Delta M_{s}=\frac{M_{t}x_{st}-M_{0}x_{s0}}{M_{0}}$$

where *M* represents the mass (kg), *x_i_* is the mass fraction of the compound *i* (kg_i_/kg_T_), being the different compounds represented by subscripts: *w* the water, and *s* the soluble solids; moreover, the subscripts *t* represent the treatment time, being 0 the initial value.

In Figure 2, it can be observed that the greatest loss of mass occurs during the first 1722 min of the OD treatment. From that moment, there was no variation in the total mass of the sample or in the mass of water. In contrast, the increase in soluble solids occurred during the first 720 min, being this content stabilized from this point.

**Figure 3:**
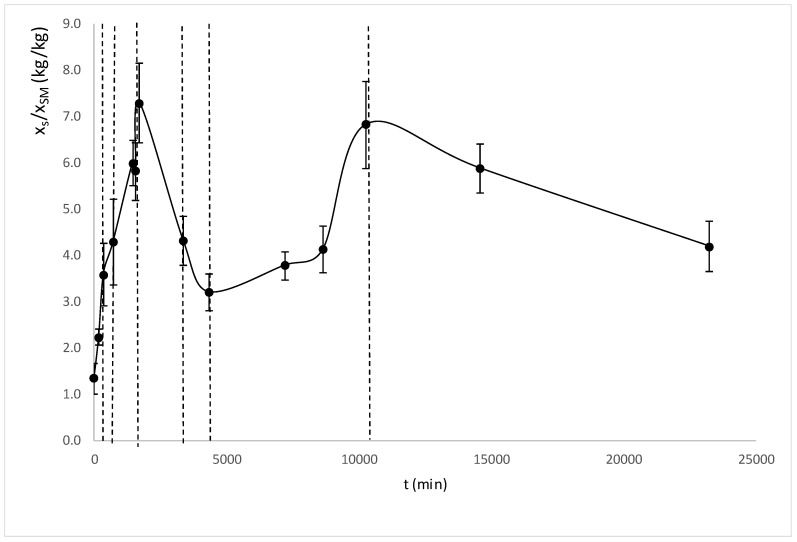
Sucrose/solid matrix relation with regard to osmotic dehydration (OD) time. Dotted lines represent the OD samples times chosen for the samples that will be dehydrated later by HAD.

The mass variation evolution does not allow us to observe correctly the chemical equilibrium that happens when the plasmolysis occurs [54]. The plasmolysis or breakdown the bonds between the protoplast and the wall precedes tissue shrinkage and swelling [56], and these phenomena change the amount of liquid phase but not its composition. For this reason, the relationship between the sucrose content and the solid matrix with respect to the treatment time is shown in Figure 3.

In Figure 3, it is possible to observe how the sucrose/solid matrix weight relation reaches a maximum (cellular plasmolysis) at 1722 min. Seven different osmodehydration treatment times were selected which correspond to seven different sucrose/solid matrix weight relations: 0 min of OD (1.33 kg_sucrose_/kg_solid matrix_), 360 min of OD (3.58 kg_sucrose_/kg_solid matrix_), 720 min of OD (4.27 kg_sucrose_/kg_solid matrix_), 1722 min of OD (7.28 kg_sucrose_/kg_solid matrix_), 3375 min of OD (4.31 kg_sucrose_/kg_solid matrix_), 4320 min of OD (3.20 kg_sucrose_/kg_solid matrix_) and 10,270 min of OD (6.82 kg_sucrose_/kg_solid matrix_). Two OD treatment times were selected before the plasmolysis in order to study the samples before the chemical equilibrium. After the plasmolysis, the samples suffered mechanical phenomena (swelling and shrinkage), which were responsible for the osmotic solution intake or outflow; from this time there were no longer diffusional driving forces. After 1722 min of OD, the samples suffered a shrinkage causing the outflow of liquid phase, and therefore the decrease in the sucrose content (decrease in the weight relation sucrose/solid matrix). Two OD treatment times were selected in this period (3375 and 4320 min of OD). After the shrinkage, the swelling phenomenon occurred, which could be observed by the increase of sucrose/solid matrix weight relation. One OD treatment time was selected in this period: 10,270 min.

**Figure 4:**
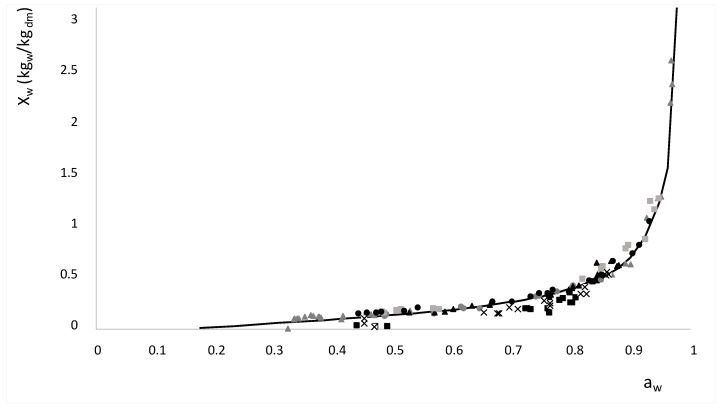
Moisture (kg water/kg dry matter) with regard to the water activity, where: (―) represents a water-sucrose solution [57], (▲) 1.33 kg_sucrose_/kg_solid matrix_, (■) 3.58 kg_sucrose_/kg_solid matrix_, (●) 4.27 kg_sucrose_/kg_solid matrix_, (▲) 7.28 kg_sucrose_/kg_solid matrix_, (x) 4.31 kg_sucrose_/kg_solid matrix_, (●) 3.20 kg_sucrose_/kg_solid matrix_ and (■) 6.82 kg_sucrose_/kg_solid matrix_.

Figure 4 shows the relationship between the water activity of samples in equilibrium (24 h after HAD treatment) and moisture expressed in dry matter (x_w_/1 − x_w_), or isotherm at 30 °C. Moreover, Figure 4 shows the isotherm of pure water/sucrose solution at 30 °C from [57], where it is possible to observe that the apple isotherm data are around the isotherm of pure solution. The range of water activity measured covers some state transitions associated with water and sucrose. Thus, it is necessary to analyze the possible transitions that occur during the HAD treatment. 

**Figure 5:**
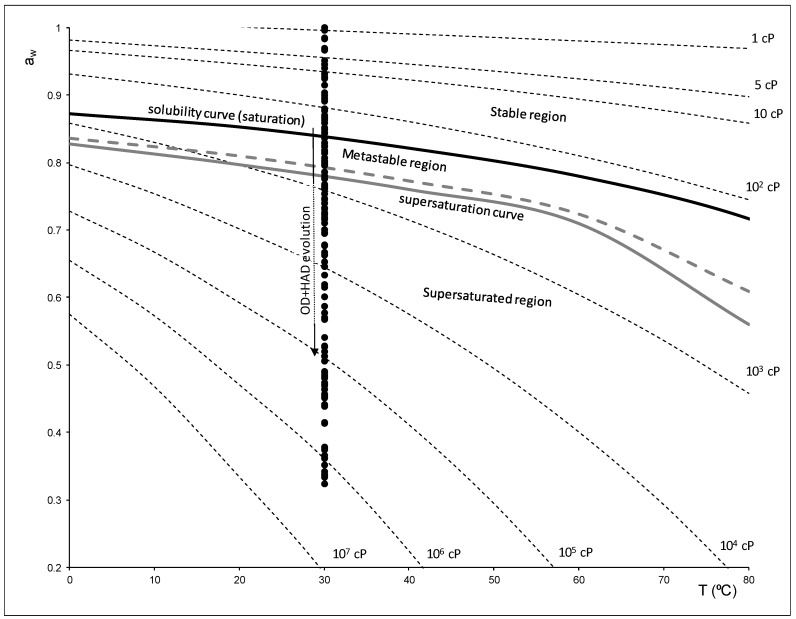
Diagram state of sucrose solution represented as water activity vs. temperature, where ( 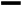) and (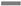) lines represent the saturation and supersaturation curves obtained from [16], (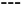) lines represent different values of viscosity obtained from Lewis (1990) [58], and (●) represent the different experimental values of samples with OD and HAD treatments.

Figure 5 shows the diagram state of the sucrose–water solution represented as water activity versus temperature. In this figure, it is possible to observe how at 30 °C the samples crossed the saturation and supersaturation curve as the viscosity of the liquid phase increased. This represents that the liquid phase analyzed could be in the stable, metastable or supersaturated region. Therefore, when developing a predictive tool for the water state during an OD or HAD treatment, it is important to predict not only the composition but also to predict the water mobility or the state of the liquid phase of the tissue being treated.

In order to develop a tool to predict the state of the liquid phase throughout the treatment of OD or HAD, the dielectric properties were analyzed in the range of microwaves, where the γ (dipolar effect) affects mainly the water molecules, generating an orientation and induction effect that was greater when the mobility of the molecules was greater.

**Figure 6:**
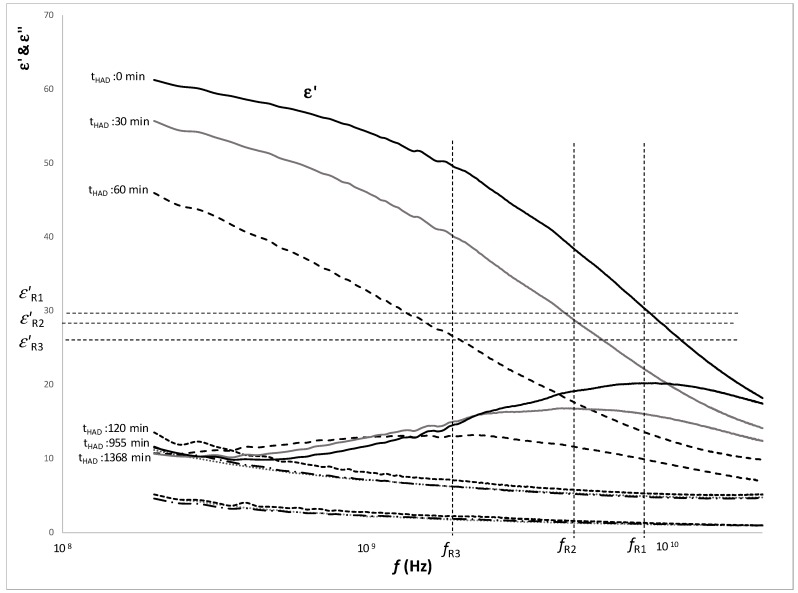
Dielectric constant and loss factor spectra of samples treated 360 min with OD and different HAD treatment times (t_HAD_), where: *f_R_* is the relaxation frequency, and sub-indices *R1, R2* and *R3* refer to the relaxation of γ-dispersion of samples dehydrated 0, 30 and 60 min, respectively.

Figure 6 shows the dielectric constant and loss factor spectra of samples treated 360 min with OD and different HAD treated times, as an example of spectra variation throughout the HAD treatment. The dielectric phenomena by orientation and induction occurred in the range of radiofrequency and microwaves. The phenomena were three, α, β and γ and occurred in a large frequency range. It is considered that each phenomenon has a maximum effect when the losses are maximum and the frequency at which the maximum effect occurs are called the relaxation frequency. In order to understand the relationship of each phenomenon with the molecules affected, it is necessary to adjust the spectrum to a model that obtains the relaxation values of each phenomenon. The Traffano-Schiffo model [20] has been applied to obtain the relaxation values in the γ-dispersion. In Figure 6, it is possible to observe some relaxation frequencies decreasing with the HAD treatment time.

The dielectric constant is the part of the permittivity that describes the orientation (electrical storage) of the water molecule. Dielectric constant is higher when the movement capacity of the water molecules is greater, as well as higher the number of water molecules is.

**Figure 7:**
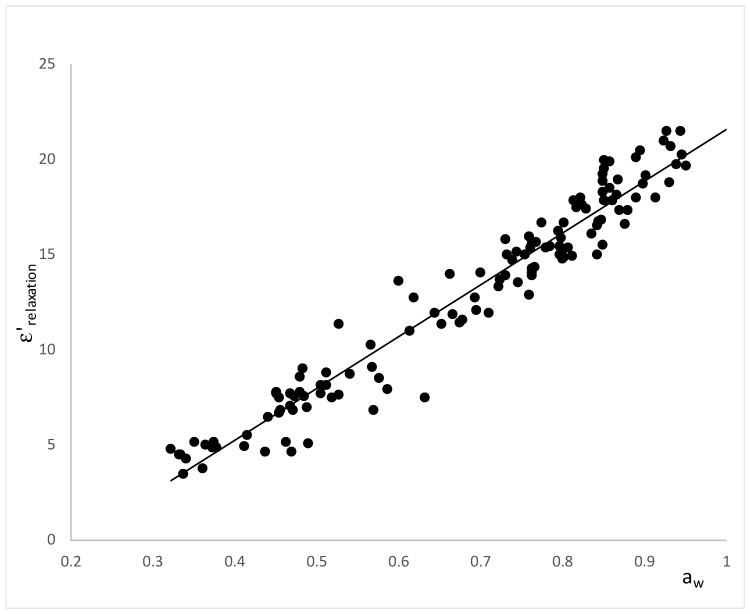
Relation between the dielectric constant at the relaxation frequency and the water activity.

Figure 7 shows the relation between the dielectric constant at relaxation frequency and the water activity, where it is possible to observe a linear relation between the dielectric constant at the relaxation frequency and the water activity, regardless of the sucrose/solid matrix weight relation, the structure changes or the rheologic transitions. Therefore, only the water mobility affected the water orientation, and thus, dielectric constant in relaxation frequency represents an excellent tool to predict the water activity but not the transitions of water/sucrose.

The linear regression obtained was (R^2^ = 0.944):

(5) $$a_{w}=\;0.0346\cdot {\varepsilon^{\prime }}_{relaxation}\;+\;0.2363.$$

The constant of the equation (0.2363), which represents the activity of water for a null dielectric constant or null water capacity for orientation, coincides with the water activity at which sucrose crystallizes (0.23) [59].

**Figure 8:**
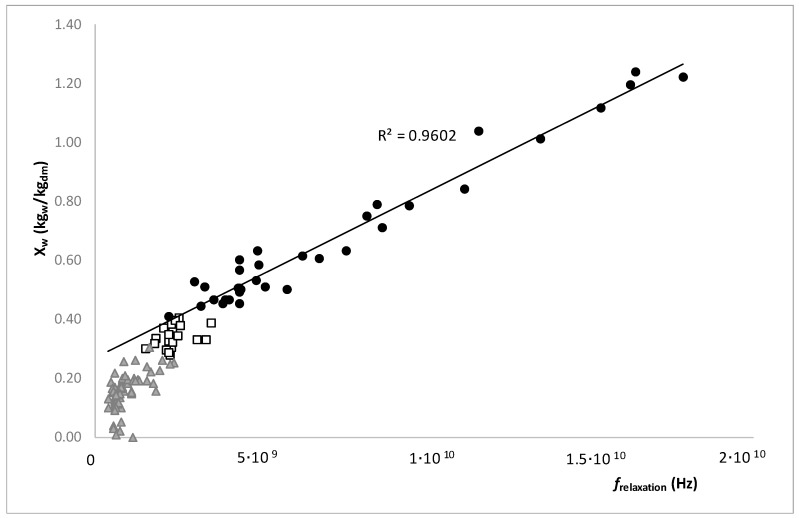
Relation between the moisture in dry basis (kg_water_/kg_dry matter_) with the relaxation frequency, where (●) represents the samples in stable region, (**□**) represents the samples in metastable region and (▲) the samples in the supersaturated region.

Figure 8 shows the relationship between the moisture in dry basis and the relaxation frequency in the different regions described in Figure 5. As it is possible to observe in this figure, only the samples that were in the stable region showed a linear relation, in this region the liquid phase had great mobility and the viscosity was low. Samples located in a metastable or supersaturated region moved away from linearity. This meant that it was possible to discriminate the regions of the state diagram of sucrose/water by analyzing the frequency of relaxation.

With the purpose of being able to use dielectric measurements in the microwave range to predict the sorption isotherm of apples in the process of OD and HAD, besides being able to correctly describe the water/sucrose state, the frequency of relaxation was compared with the loss factor.

**Figure 9:**
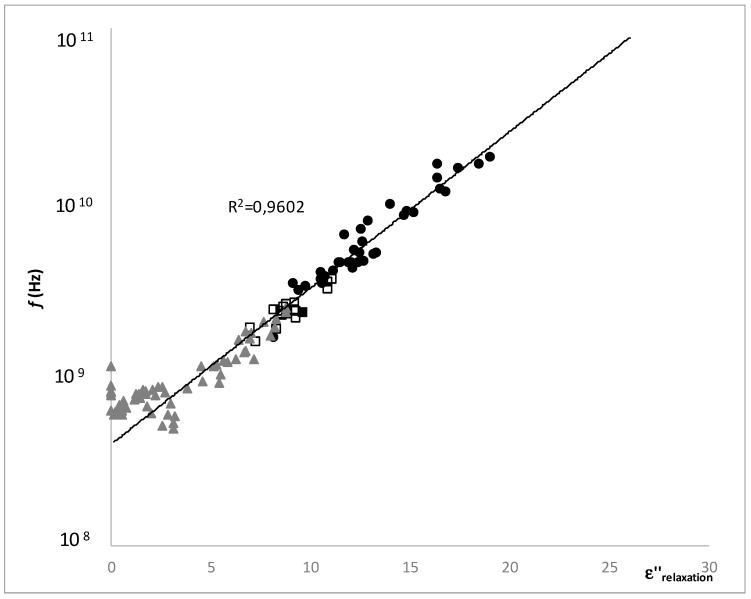
Semi-logarithmic relation between the relaxation frequency and the loss factor at relaxation frequency, where (●) represents the samples in stable region, (**□**) represents the samples in metastable region, (▲) the samples in the supersaturated region and (—) represents the linear regression of all data together.

Figure 9 shows the semilogarithmic relationship of the relaxation frequency and the loss factor at relaxation frequency, segregated according to the stability region. As it is possible to observe the relation between both was linear in all the regions, and did not allow the separation. Nevertheless, at the very low mobility zone in the supersaturated region, new behavior appeared because the relaxation frequency remained constant while the loss factor was reduced, this might be due to the vitrification process (glass transition).

## 4. Discussion

The treatment of OD produces compositional and structural transformations within the tissue and the HAD treatments produce water losses, structural transformations and transitions in the liquid phase. In order to analyze the two treatments it is necessary to include several critical points of both processes such as plasmolysis, compression/relaxation phenomena or saturation and supersaturation of the liquid phase. In Figure 3, it is possible to observe how the sucrose/solid matrix weight relation reaches a maximum (plasmolysis) at 1722 min.

Samples treated with OD and HAD, plasmolyzed or not, cross the saturation and supersaturation curve as the viscosity of the liquid phase increases. This represents that the liquid phase of samples analyzed could be in the stable, metastable or supersaturated region. Therefore, when developing a predictive tool for describing the water state during an OD or HAD treatment, it is important to predict not only the composition but also to predict the water mobility or the state of the liquid phase of the tissue being treated. 

The Traffano-Schiffo model allows obtaining the relaxation values in the microwave range, it allows us to represent the dielectric constant at relaxation frequency versus the water activity, where it is possible to observe a linear relation between both variables. Therefore, only the water mobility affects to the water orientation, and thus, dielectric constant in relaxation frequency represents an excellent tool to predict the water activity but not the transitions of water/sucrose. Moreover, the good fit of the data is shown in the good values of the correlation coefficient and also in the coincidence between the constant of the equation and the water activity at which sucrose crystallizes (0.23).

The relationship between the moisture in the dry basis and the relaxation frequency in the different regions described shows that only the samples that are in the stable region have a linear relation. In this region, the liquid phase has great mobility and the viscosity is low. Samples located in a metastable or supersaturated region move away from linearity. This means that it is possible to discriminate the regions of the state diagram of sucrose/water by analyzing the frequency of relaxation.

The relaxation frequency and the loss factor at the relaxation frequency had a semilogarithmic, relationship in all the regions, and did not allow the separation. Nevertheless, at the very low mobility zone in the supersaturated region, new behavior appeared because the relaxation frequency remained constant while the loss factor was reduced, this might be due to the vitrification process (glass transition).

In the dielectric analysis in γ-dispersion, samples before and after the OD plasmolysis showed no differences, therefore, in this case of parenchymatic tissue, the effect of membrane plasmolysis had no effect in the water orientation or induction. Therefore, in similar fruit tissue, with high quantity of parenchymatic tissue and low vascular tissue, the behavior could be expected to be similar. 

## 5. Conclusions

The use of specific frequencies of the dielectric properties in γ-dispersion did not allow us to correctly analyze the mobility of water and it makes it necessary to determine the dielectric properties at the relaxation frequency. It was demonstrated that the dielectric constant (at relaxation frequency) was linearly related to the water activity and not to the moisture, it means that it was affected by water mobility and therefore by the structure. In addition, it was possible to determine sucrose supersaturation processes by analyzing the relaxation frequency, which depends on the deformation of the water molecule. Therefore, it was demonstrated that the use of dielectric properties in γ-dispersion at relaxation frequency allowed us not only to monitor the OD and HAD processes of the apple candying, but also to predict the supersaturation state of the liquid phase until vitrification.
